# Direct correlation of MRI with histopathology in pediatric renal tumors through the use of a patient-specific 3-D-printed cutting guide: a feasibility study

**DOI:** 10.1007/s00247-022-05476-7

**Published:** 2022-08-30

**Authors:** Justine N. van der Beek, Matthijs Fitski, Ronald R. de Krijger, Marc H. W. A. Wijnen, Marry M. van den Heuvel-Eibrink, Marijn A. Vermeulen, Alida F. W. van der Steeg, Annemieke S. Littooij

**Affiliations:** 1 Princess Máxima Center for Pediatric Oncology, Utrecht, The Netherlands; 2 Department of Radiology and Nuclear Medicine, University Medical Center Utrecht, Heidelberglaan 100, 3584 CX Utrecht, The Netherlands; 3 Department of Pathology, University Medical Center Utrecht, Utrecht, The Netherlands

**Keywords:** Children, Kidney, Magnetic resonance imaging, Neoplasm, Nephroblastoma, Pathology, Three-dimensional printing, Wilms tumor

## Abstract

**Background:**

Pediatric renal tumors are often heterogeneous lesions with variable regions of distinct histopathology. Direct comparison between in vivo imaging and ex vivo histopathology might be useful for identification of discriminating imaging features.

**Objective:**

This feasibility study explored the use of a patient-specific three-dimensional (3D)-printed cutting guide to ensure correct alignment (orientation and slice thickness) between magnetic resonance imaging (MRI) and histopathology.

**Materials and methods:**

Before total nephrectomy, a patient-specific cutting guide based on each patient’s preoperative renal MRI was generated and 3-D printed, to enable consistent transverse orientation of the histological specimen slices with MRI slices. This was expected to result in macroscopic slices of 5 mm each. The feasibility of the technique was determined qualitatively, through questionnaires administered to involved experts, and quantitatively, based on structured measurements including overlap calculation using the dice similarity coefficient.

**Results:**

The cutting guide was used in eight Wilms tumor patients receiving a total nephrectomy, after preoperative chemotherapy. The median age at diagnosis was 50 months (range: 4–100 months). The positioning and slicing of the specimens were rated overall as easy and the median macroscopic slice thickness of each specimen ranged from 5 to 6 mm. Tumor consistency strongly influenced the practical application of the cutting guide. Digital correlation of a total of 32 slices resulted in a median dice similarity coefficient of 0.912 (range: 0.530–0.960).

**Conclusion:**

We report the feasibility of a patient-specific 3-D-printed MRI-based cutting guide for pediatric renal tumors, allowing improvement of the correlation of MRI and histopathology in future studies.

**Supplementary Information:**

The online version contains supplementary material available at 10.1007/s00247-022-05476-7.

## Introduction

Renal tumors account for 5–6% of all pediatric malignancies [1]. Nephroblastoma (Wilms tumor) is the most common type of childhood kidney cancer. In the International Society of Pediatric Oncology – Renal Tumor Study Group (SIOP-RTSG) 2016 UMBRELLA protocol, treatment of children with renal tumors starts with preoperative chemotherapy and histopathology is commonly confirmed only after surgery [2–4]. Imaging before surgery might play a fundamental role for the noninvasive discrimination of tumor subtypes as an in vivo biomarker. This might impact early treatment decisions, which could lead to enhanced survival as well as reduced treatment-related toxicity.

Computed tomography (CT) and magnetic resonance imaging (MRI) have shown to be equivalently adequate for loco-regional staging of pediatric renal tumors. Within the SIOP-RTSG 2016 UMBRELLA protocol, MRI is the preferred imaging modality, providing high soft-tissue contrast as well as quantitative information such as diffusion-weighted imaging (DWI) without the use of ionizing radiation [5, 6]. Recent studies have identified an association between apparent diffusion coefficient (ADC) values and histopathological findings in renal tumors [7–12].

Pediatric renal tumors are commonly large heterogeneous tumors, especially in the case of nephroblastomas, which show variable portions of histopathological components (epithelial, stromal and/or blastemal cells, with or without anaplasia). This contrasts with the adult population, in whom more homogeneous renal tumors are often the case. Previous studies focusing on DWI in pediatric renal tumors often used whole tumor ADC values, which may possibly obscure underlying specific correlations of histopathological subtypes [11, 13, 14].

To further improve the noninvasive discrimination of pediatric renal tumor subtypes based on objective radiologic indicators, a direct visual correlation between in vivo imaging and ex vivo histopathology is required [15]. Previously, correlating histopathological and radiology data was attempted after freehand slicing of the specimen, resulting in macroscopic slices that did not correspond with the orientation and slice thickness of the cross-sectional imaging [7, 8]. For this reason, three-dimensional (3-D)-printed cutting guides, ensuring identical orientation and thickness of slices obtained from gross specimens and from imaging, have been developed for oncological diagnoses such as prostate and breast cancer [16–19]. Recently, this technique has been used for renal tumors in adults but has not yet been applied to pediatric renal tumors, which are often very large lesions with areas of hemorrhage and/or necrosis [20–22]. The use of 3-D printing technology has enabled assessment of imaging features and histopathological data of the same regions of tumors, while also facilitating the pathologist in the specimen slicing process [19]. Overlap calculation to determine the degree of correlation of slices is often done based on the dice similarity coefficient, which is a spatial overlap index in which 0 means no overlap and 1 is considered perfect overlap [21]. In this context, correlation is used as a term to describe the matching of histopathology and radiologic imaging and is not used as a statistical concept.

Establishing this direct comparison of radiology and histopathology in the pediatric population could allow identification of MRI-DWI characteristics for the discrimination of clinically relevant histological subtypes. This could improve MRI-based assessment of renal tumors at the time of diagnosis and prediction of their response to preoperative therapy [7, 8, 13, 23–25]. Therefore, we have designed and implemented a patient-specific preoperative 3-D-printed cutting guide for children with renal tumors in this feasibility study. We aimed to determine the value of the cutting guide for direct correlation between MRI and histopathology in this specific patient population. The aim is to develop this technique for further use in larger patient cohorts to define radiological characteristics for the potential use of developing MRI-DWI as a noninvasive biomarker.

## Materials and methods

### Patients

In this prospective feasibility study, eight consecutive patients diagnosed at our center with a renal tumor between October 2020 and April 2021 were included. Inclusion criteria were pediatric age (< 18 years), radiologically proven renal tumors, informed consent and inclusion in the SIOP-RTSG 2016 UMBRELLA protocol, complete MRI protocol including DWI before surgery, availability of a patient-specific 3-D-printed cutting guide based on the preoperative MRI scan and availability of the tumor specimen for histopathological assessment. Patients were excluded in case of nephron-sparing surgery. The institutional ethical board approved this prospective feasibility study and waived the requirement for a separate informed consent since this study and MRI were embedded in the SIOP-RTSG 2016 UMBRELLA study with no additional burden for the patient.

### MRI acquisition and image analysis

All abdominal MRI examinations including DWI were performed in our institution on a 1.5-T scanner (Ingenia; Philips Medical Systems, Best, The Netherlands), following the standard of care SIOP-RTSG MRI protocol, including DWI described by Watson et al. [26]. Children were awake, sedated or under general anesthesia depending on their ability to cooperate, according to standard of care procedures. All children were screened for contraindications for MRI, intravenous contrast agents and intravenous hyoscine butylbromide. Gadobutrol (Gadovist; Bayer B.V., Leverkusen, Germany) was administered intravenously at a dose of 0.1 ml/kg body weight and 0.4 mg/kg body weight of hyoscine butylbromide (Buscopan; Sanofi, Paris, France) was administered intravenously (with a maximum of 10 mg in children ages 6 years and older, and a maximum of 5 mg in children younger than 6 years) to reduce peristaltic artifacts.

### Tumor and kidney segmentation

For the tumor and kidney segmentation, the post-contrast T1-weighted sequence was used (Table 1). The anonymized fat-suppressed post-contrast T1-weighted MRI sequence was delineated in the open-source software 3DSlicer 4.11.20200930 by two experienced users (M.F., clinical technologist, with 3 years of experience; J.N.vdB., medical doctor, with 3 years of experience) [27]. A semiautomatic 3-D region growing algorithm was used to segment the tumor and kidney based on manual annotations. The results were smoothed with an averaging filter and manually confirmed.

**Table 1:** Scan parameters at 1.5-T magnetic resonance imaging used for the patient-specific 3-D-printed cutting guide

Parameters	T1-W pre-/post-contrast	DWI
Pulse sequence	2-D ultrafast spoiled gradient echo with fat suppression	2-D single-shot spin echo with spectral fat saturation
Repetition time (ms)	5.5	2,607
Echo time (ms)	2.7	73
Slice thickness (mm)	3	5
Slice orientation	Axial	Axial
Echo train length	60	35
Slicing gap	1	0
Acquisition matrix	233 × 233	88 × 70
b-values	-	At least 0, 100 and 1,000

*DWI* diffusion-weighted imaging, *mm* millimeters, *ms* milliseconds, *W* weighted

### 3-D printing of a patient-specific cutting guide

**Fig. 1:**
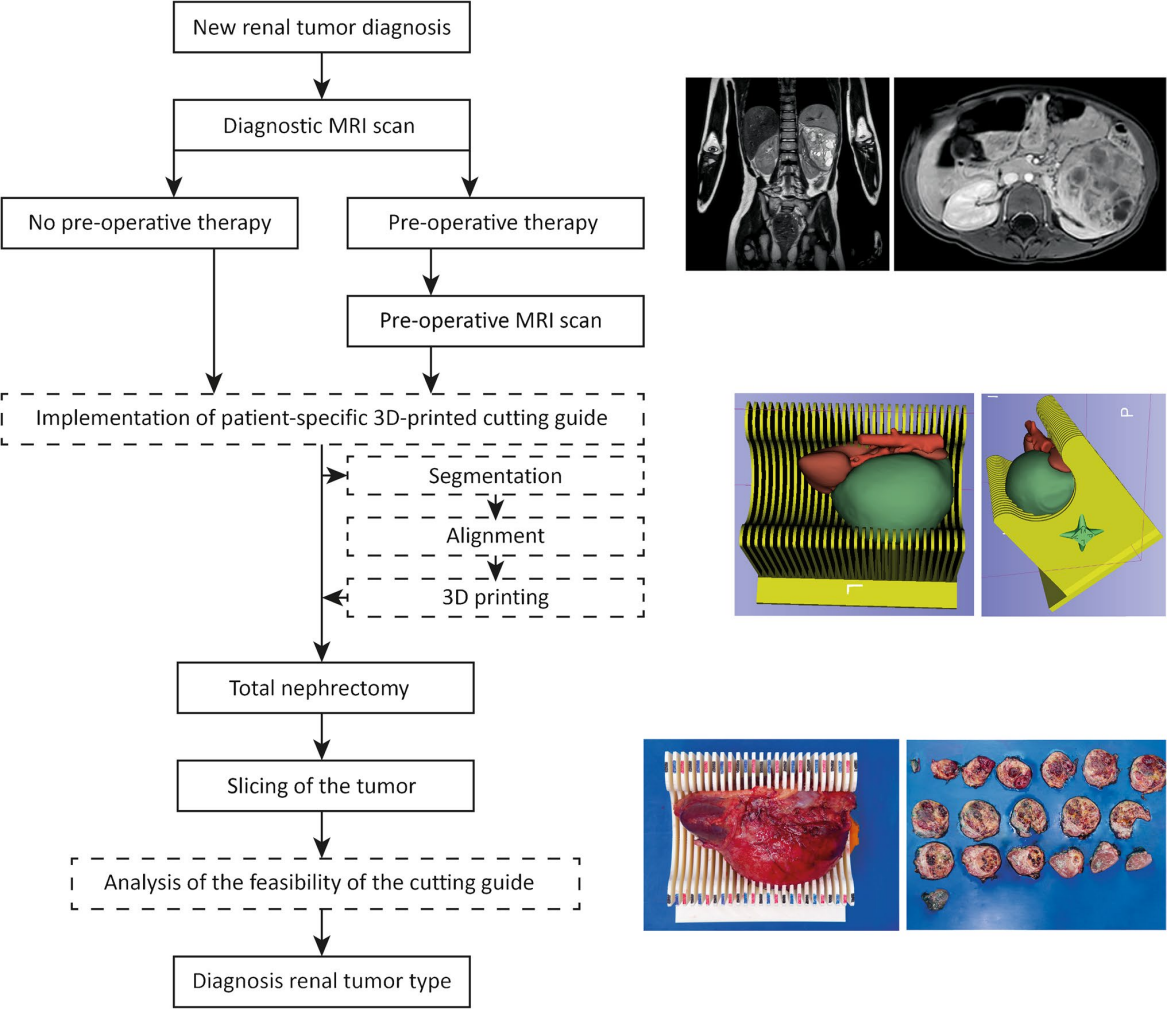
Implementation of the patient-specific 3-D-printed cutting guide in the clinical workflow for pediatric renal tumors. ─── clinical care, --- implementation of the cutting guide

After segmentation of the tumor and kidney, the cutting guide was digitally orientated to match the direction of the slices on the DWI-MRI scan. The tumor/kidney model was always digitally orientated with the cranial side of the tumor to the right of the cutting guide, facilitating the slicing process of the specimen from cranial to caudal (Fig. 1). Furthermore, the renal hilum was positioned on the open side of the cutting guide as central as possible, led by a lateral digital orientation for optimal fitting in the base of the cutting guide.

To create the patient-specific cutting guide, the shapes of the kidney and tumor were digitally extracted from the model using computer-aided design software (Fusion 360; Autodesk, Inc., San Rafael, CA). The resulting cutting guide was digitally prepared in Cura 4.8.0 (Ultimaker, Utrecht, The Netherlands, RRID:SCR_018898) with default printer settings for 3-D printing with a fused filament fabrication 3-D printer (Ultimaker S5; Ultimaker, Utrecht, The Netherlands) using polylactic acid (Fig. 1). The foreseen orientation of the specimen was indicated on the cutting guide with a compass. The cutting guide barriers were alternately colored to support the pathologist in correctly positioning the knife. Moreover, these colors allowed for the repositioning of two movable support barriers to keep the specimen positioned during the slicing process *(*Fig. 1, Online Supplementary Material 1).

### Workflow

The patient-specific 3-D-printed cutting guide was implemented in the clinical standard of care workflow for pediatric patients with a renal tumor (Fig. 1). Preoperative chemotherapy according to the SIOP-RTSG 2016 UMBRELLA protocol consisted of 4 weeks of vincristine/actinomycin-D (stage I-III) or 6 weeks of vincristine/actinomycin-D/doxorubicin (stage IV/V). The cutting guide was designed before surgery based on the most recent MRI scan after preoperative chemotherapy. The specimen was positioned in the cutting guide by the pediatric surgeons (M.H.W.A.W. with 23 years of experience; A.F.W.vd.S. with 13 years of experience) directly after surgical removal. This process was guided by the lead investigators (M.F. and J.N.vd.B.) (Fig. 1). After transportation, the specimen was only temporarily removed from the cutting guide by the pathologist (R.R.d.K., with 23 years of experience; M.A.V., with 5 years of experience) for inking, photographing and measuring according to SIOP-RTSG 2016 UMBRELLA protocol. The specimen was thereafter repositioned by the pathologist, under guidance of the lead investigators.

The pathologist used the cutting guide with the aim of producing macroscopic slices of 5 mm each, corresponding with the slice thickness and the orientation of the DWI sequence. The slices were positioned according to the appearance on MRI from cranial to caudal (Fig. 1).

### Qualitative and statistical analysis

The feasibility of the application of the patient-specific 3-D-printed cutting guide was assessed both qualitatively and quantitatively. A questionnaire distributed among involved experts focused on the feasibility of the workflow, from the positioning of the specimen in the cutting guide in the operating room until the slicing of the tumor was completed. The ease of use and process efficiency was assessed based on 5-point Likert scales, ranging from very difficult to very easy and from definitely not effective to very effective, with a field to leave comments (Online Supplementary Material 2).

Quantitatively, we measured the slice thickness, performed overlap calculations using the dice similarity coefficient and matched the level of anatomical landmarks in the tumor. The thickness of all slices was measured on two opposite sides using the longest diameter as axis. The macroscopic slices were photographed, and the tumor tissue was manually delineated in 3DSlicer in four representative slices for each patient. This segmentation of the macroscopic photo was scaled and rotated with a similarity registration algorithm to match with the segmentation of the corresponding DWI slice and fat-suppressed post-contrast T1-weighted slice. Then, the dice similarity coefficient determined the overlap of the segmentation and macroscopic slice. For this purpose, a dice similarity coefficient of 0.800 was considered a good overlap. Slices containing anatomical landmarks, such as the renal hilum and the upper and lower pole calyces, preoperatively identified by the radiologist (A.S.L., pediatric radiologist, with 18 years of experience) on MRI, were postoperatively identified by the pathologist after slicing the specimen, blinded to the radiologist’s assessment.

## Results

### Patient characteristics

Eight patients with a renal tumor were included (Table 2). The median age at diagnosis was 50 months (range: 4–100 months) and 50.0% of the patients were male. All patients were histologically diagnosed with a nephroblastoma. Six patients had stage I, 1 patient stage II and 1 patient stage III disease. All patients had received 4 weeks of preoperative chemotherapy with vincristine and actinomycin-D. The median tumor volume after preoperative therapy was 252 cm^3^ (range: 2–1250 cm^3^) (Table 3).

**Table 2:** Baseline characteristics

Patient no	Age at diagnosis *(months)*	Sex	Disease stage^a^	Preoperative treatment	Tumor type^b^	Nephrogenic rest(s) / nephroblastomatosis
1	100	M	I	VA, 4 weeks	WT, regressive	No
2	4	M	I	VA, 4 weeks	WT, regressive	No
3	7	M	I	VA, 4 weeks	WT, stromal	No
4	76	F	II	VA, 4 weeks	WT, stromal	Yes
5	50	F	I	VA, 4 weeks	WT, mixed	Yes
6	30	F	I	VA, 4 weeks	WT, regressive	No
7	80	F	III	VA, 4 weeks	WT, regressive	Yes
8	50	M	I	VA, 4 weeks	WT, mixed^c^	Yes

^a^ Disease stage defined according to the TNM-classification system defined in the International Society of Pediatric Oncology – Renal Tumor Study Group (SIOP-RTSG) 2016 UMBRELLA classification

^b^ Tumor type defined after surgery, following the SIOP-RTSG 2016 UMBRELLA protocol

^c^ Mixed type nephroblastoma with diffuse anaplasia

*A* actinomycin-D, *F* female, *M* male, *no.* number, *V* vincristine, *WT* Wilms tumor (nephroblastoma)

**Table 3:** Tumor- and patient-specific 3-D-printed cutting guide characteristics

Patient no	Tumor volume at diagnosis (cm^3^)	Tumor volume after preoperative therapy (cm^3^)	Visual estimation of enhancing tumor tissue after preoperative therapy (%)	Specimen weight after TN (grams)	Time from preoperative MRI to TN (days)	Time needed for design of the cutting guide (minutes)	Time needed for printing the cutting guide (minutes)	Cost of printing the cutting guide (euros)
1	857	190	30	420	7	240	2,160	24.84
2	10	2	95	39	5	75	1,440	13.94
3	1,046	1,250	40	1,473	5	160	2,340	38.07
4	912^a^	272	60	404	2	110	1,124	12.13
5	708	231	65	336	7	105	1,680	14.58
6	543	386	2	566	3	50	1,680	22.23
7	118 *3.3*^b^	9 *1*^b^	5 *0*^b^	182	5	90	1,215	15.58
8	1,481	633	50	675	5	90	2,340	31.28

^a^ No diagnostic magnetic resonance imaging (MRI) scan available, so volume based on computed tomography (CT) scan at diagnosis

^b^ Tumor consisting of two separate lesions, with the smallest lesion being a nephrogenic rest with a high risk for partial voluming effect, and therefore not included in the median overall tumor volume

*cm* centimeter, *no.* number, *TN* total nephrectomy

### Patient-specific 3-D-printed cutting guide details

Following preoperative chemotherapy, a patient-specific cutting guide was designed for each individual patient based on the preoperative MRI scan, with a median of 5 days (range: 2–7 days) before the total nephrectomy (Fig. 1, Table 3, Online Supplementary Material 1). The manual segmentation and cutting guide design took a median time of 98 min (range: 50–240 min), whereas 3-D printing of the cutting guide took a median of 28 h (range: 18.7–39.0 h). The median cost of the material for printing a cutting guide was €18.90 (range: €12.13–38.07) (Table 3).

### Feasibility of workflow and cutting process

The placement of the specimen inside the cutting guide in the operating room was performed under guidance of the surgeon in all cases and rated overall as 5 out of 5 (very effective, range: 3–5) (Online Supplementary Material 3). Maintaining the position of the specimen in the cutting guide during transportation to the diagnostic laboratory was uncomplicated. Repositioning of the specimen after the standard clinical pathological workflow procedures (under the guidance of the lead investigator) was rated overall as 4.5 out of 5 (easy/very easy, range: 4–5). Slicing of the specimen in the cutting guide was rated as 4 out of 5 (easy, range: 2–5) (Online Supplementary Material 3). The most frequently reported challenge during the pathological slicing was the weak consistency of the specimen in tumors with gross necrotic tissue, cysts and/or hemorrhage. This resulted in discrepancies in the slicing results. The estimated additional time needed by the pathologist for the use of the cutting guide in individual cases was approximately 30 min.

The quality of the cutting guide was deemed poor by the pathologist for two inclusions due to inadequate 3-D printing settings, caused by the decision to shorten the 3-D printing time because of a limited availability of time before surgery (Table 3). The 3-D printing settings were improved during the study, resulting in a stable cutting guide without increasing printing time (Online Supplementary Material 4).

### Quantitative analysis of comparability between imaging and histopathology

**Fig. 2:**
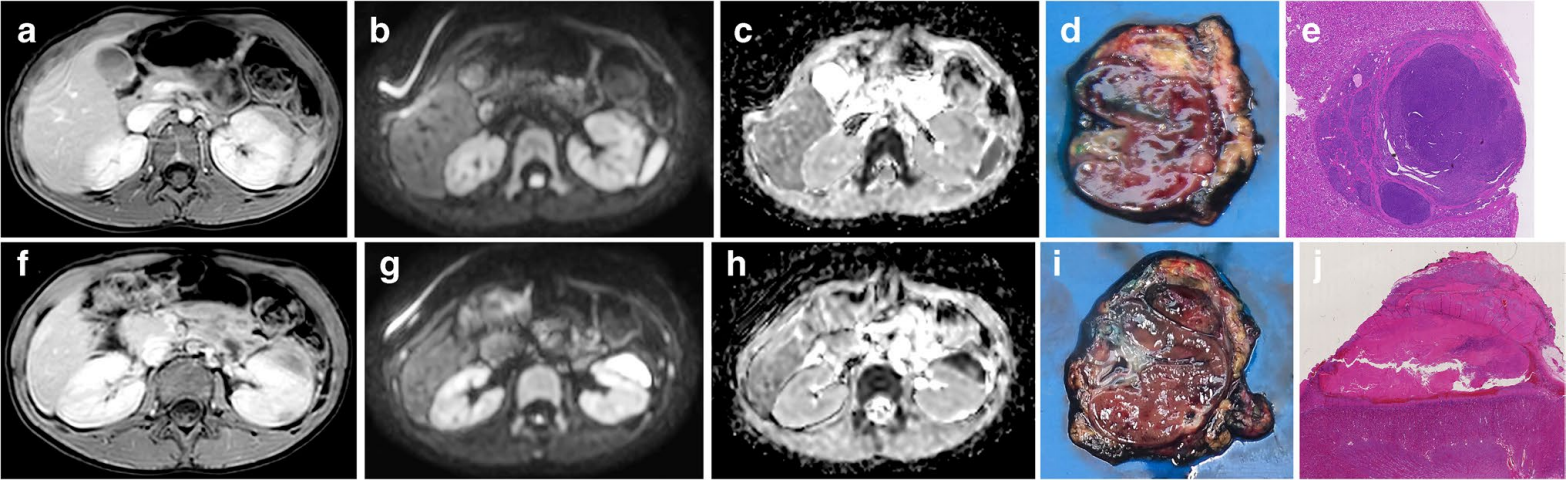
An example of the visual matching of radiologic slices and histopathological macroscopy and microscopy. An 80-month-old girl with a left regressive Wilms tumor (98% therapy effect) and nephrogenic rest after 4 weeks of preoperative chemotherapy. Axial slices at two levels (**a-e** and **f-j**) are shown on post-contrast T1-weighted magnetic resonance imaging (MRI) (**a, f**), diffusion-weighted MRI b1000 (**b, g**), apparent diffusion coefficient MRI (**c, h**), macroscopic histopathology (**d, i**) and microscopic histopathology with hematoxylin and eosin stain (**e, j**). Images (**a-c**) show a nephrogenic rest that was directly correlated to the macroscopy and microscopy (**d, e,** *1.0/40* × *magnification*). In (**f–h),** the ventral tumor component was correlated with histopathology (**i, j,** *0.4/44* × *magnification*)

This study focused on the feasibility of the technique. Nevertheless, a direct visual correlation of MRI and histopathology slices was attempted (Fig. 2). This was not further explored in this feasibility study.

#### Slice thickness

The use of the patient-specific 3-D-printed cutting guide resulted in a median slice thickness per patient ranging from 5 to 6 mm. The median difference per patient between opposite sides of the slice ranged from 0 to 2 mm. In seven patients, there was a discrepancy in the number of target slices based on the DWI and the cutting guide and the resulting number of slices after use of the cutting guide. The discrepancy in the number of slices ranged from 59.4% to 90.3% (Table 4). Necrotic, hemorrhagic and cystic areas, typically nonenhancing on MRI, were the most important causes of this discrepancy (Table 3). This resulted in slices thicker than the intended 5 mm and incomplete slices due to distortion of the specimen in the cutting guide. The discrepancy decreased with the rising number of included patients.

**Table 4:** Characteristics of the slices and the slicing process

Patient No.	Median slice thickness^a^ (mm)	Range slice thickness^a^ (mm)	Median opposite difference in slice thickness^a^ (mm)	No. of target slices	No. of resulted slices	Average DSC	Range DSC	Number of correlating anatomical landmarks / range of discrepancy
1	5	1–18	2	29	23	0.862	0.760—0.930	1/2
2	6	2–11	1	17	14	0.861	0.812—0.914	3/4
3	5	2–18	2	32	19	0.912	0.899—0.924	4/4
4	5	1–10	1	22	19	0.881	0.799—0.943	2/4
5	5	1–8	0.5	23	20	0.928	0.910—0.940	4/4
6	5	1–9	1.5	24	20	0.925	0.880—0.960	2/2
7	5	3–8	0	20	20	0.735	0.530—0.880^b^	3/5
8	5	2–8	1	31	28	0.958	0.950—0.960	3/4

^a^ Measurement of histopathological slices after the slicing process

^b^ Slices with two lesions, leading to problematic calculation because of difficulties matching the two separate lesions after rescaling

*DSC* dice similarity coefficient, *mm* millimeters, *No.* number

#### Overlap calculation

The average dice similarity coefficient per patient ranged from 0.735 to 0.958 with a median overall dice similarity coefficient of 0.912 (range: 0.530–0.960) (Table 4). The tumor in patient 7 consisted of two separate lesions, which led to inaccurate digital matching of the regions of interest between histopathology and MRI, resulting in coefficients down to 0.530 (Table 4). When excluding this patient from the analysis, the median overall dice similarity coefficient was 0.920 (range: 0.740–0.960).

#### Anatomical landmarks

Given the observed discrepancy in the number of slices between DWI-MRI and histopathology, the levels of corresponding anatomical landmarks were identified in a range of slices, both macroscopically and from imaging. This resulted in a total of 22 out of 29 anatomical landmarks for all patients that had corresponding levels in the DWI and histopathological slices (Table 4).

## Discussion

This study implemented a patient-specific 3-D-printed MRI-based cutting guide in the clinical care for pediatric renal tumors, showing the feasibility of the technique for the direct correlation of pathology and radiology. Previous efforts at correlating histopathological and radiology data for pediatric renal tumors were mainly attempted after freehand slicing of the specimen and focused on median whole tumor ADC values [8, 28]. Freehand slicing hampers the orientation of the kidney and tumor in the abdomen on MRI and results in heterogeneity in macroscopic slice thickness. Both introduce a higher risk of interpretation bias when matching histopathology with MRI [28, 29]. However, using the patient-specific cutting guide developed in this study, the median thickness per slice was invariably 5–6 mm, corresponding to the slice thickness of the DWI-MRI. This consistency will allow direct correlation of ADC values to specific histopathology findings in multiple slices per patient, to further investigate the potential role of MRI as a noninvasive biomarker. In this way, the proposed method carries the potential to better address the intra-tumor heterogeneity of pediatric renal tumors and to identify specific viable tumor components without compromising the current clinical workflow [15, 30, 31].

Although there is increasing knowledge about semiautomatic design of 3-D models and cutting guides for renal tumors, the preferred method is often still strongly dependent on manual segmentation [22, 32]. The mean time for segmentation of the MRI and design of the cutting guide was 115 min for an experienced technician in the current study, compared to 10–25 min for an automated design with manual confirmation in a recent study on adult patients with renal cell carcinoma [22]. Manual segmentation of 14 nephroblastomas including veins, arteries and both kidneys took 8.6 h [21]. The mean printing time was 29.1 h, which seems to be somewhat longer than reported in adult renal tumor series, possibly because of the potential larger size of pediatric renal tumors and printing technique [20]. These required time spans are especially important when taking the available time to design and print the cutting guide (time between preoperative MRI and total nephrectomy) into consideration. Future studies should also focus on new automated design techniques based on artificial intelligence tools, for instance, to automate the segmentation process.

The qualitative and quantitative measures showed satisfactory results. Although a potential bias toward a perfect overlap needs to be taken into consideration when repositioning the regions of interest based on scaling and orientation in ovoid and round tumors, the median dice similarity coefficient for all patients in our feasibility study was appropriately high. This indicates an excellent spatial overlap of the tumor [22, 33]. The range of the dice similarity coefficient within patients as well as within slices was primarily caused by deformation of certain specimens during slicing, usually those with low consistency (for instance, cystic tumors). This matter could not be resolved by optimizing the design of the cutting guide or its implementation in the workflow, which makes a cutoff value for an acceptable dice similarity coefficient an important inclusion criterion for direct correlation.

The additional time needed by the team involved was considered fair and the observed decreasing discrepancy with the rising number of included patients can possibly be explained by the increasing experience of the pathologists with the patient-specific cutting guide. The current patient-specific 3-D-printed cutting guide can easily be integrated in the workflow without compromising the standard of care in our specialized center and the proposed technique was found feasible in a heterogeneous series of eight pediatric patients.

Although the results show effective application of the cutting guide, several challenges were encountered. During the study, inferior results were achieved when, due to a limited period between the preoperative MRI and the total nephrectomy, the 3-D printing time was shortened by decreasing the line width of the 3-D printing material. Given that this caused less stable cutting guides, the 3-D printing settings were optimized resulting in more compact material to achieve a stable cutting guide, thus enabling higher printing quality without increasing the printing time (Online Supplementary Material 4). Furthermore, the overall thickness of the slices resulting from each specimen was not exactly 5 mm. This automatically resulted in less slices than based on the DWI-MRI scan. Since the slicing process is particularly dependent on the consistency and composition of the tumor, large, regressive and cystic tumors can be a challenge for optimal use of the cutting guide. These characteristics are often seen in pediatric renal tumors, especially after preoperative chemotherapy. Dwivedi et al. [20] also reported potential difficulties for the correlation of tumors with cystic components, due to collapse of the tumor after sectioning caused by leakage of fluid content. Nevertheless, correction for this discrepancy resulted in correct matching of anatomical landmarks. Also, previous studies have accepted rather wide ranges of slice thickness [29, 34]. Concerning the fitting of the tumor in the cutting guide, factors such as perinephric fat, deformation and reduced pressure on the specimen compared to the intra-abdominal situation can cause certain discrepancies between the tumor and kidney model and the actual specimen [20, 22, 35]. Although we did not encounter any difficulties concerning the fitting of the tumor, a certain inaccuracy should always be taken into consideration. This positioning variability inherent to our workflow necessitates visual assessment of the correlation. Crispin-Ortuzar et al. [22] used anatomical anchor points to standardize this fitting. However, this required reslicing of the 3-D MRI based on the orientation of the anatomical points, which interferes with the workflow of the pediatric radiologist. Therefore, we did not implement this approach. 

Based on our experience and previous studies, we propose the exclusion of a sliced specimen for analysis when the number of slices is < 75% of the target number based on DWI, or when the slice with the largest representative part of the tumor shows a dice similarity coefficient of < 0.800 [15, 21]. Furthermore, preoperative chemotherapy between the preoperative MRI and total nephrectomy or  > 14 days between preoperative MRI and total nephrectomy, which may occur in rare specific cases, may induce tissue changes that can cause suboptimal fitting and may obscure valid correlation between tissue and imaging. Finally, visual assessment of the best corresponding slices remains inevitable.

In future prospective studies, the cutting guide will be used in our center for all children undergoing a total nephrectomy for renal tumors who meet our defined criteria. Further developments on the ability to identify histopathological subtypes might contribute to more personalized treatment approaches, especially in aggressive tumor subtypes, such as diffuse anaplastic and blastemal predominant nephroblastomas [21, 23, 24, 36–38].

## Conclusion

We report the feasibility of a 3-D-printed patient-specific MRI-based cutting guide for pediatric renal tumors, with the goal of improving the correlation between histopathology and MRI for a more specific use of MRI-DWI to define characteristics for the discrimination of pediatric renal tumor types in future studies.

## Supplementary Information

Below is the link to the electronic supplementary material.

Supplementary file1 (DOCX 652 kb)

Supplementary file2 (DOCX 54.1 kb)

Supplementary file3 (DOCX 15.3 kb)

Supplementary file4 (DOCX 530 kb)
