# Supplementary material for: Direct correlation of MRI with histopathology in pediatric renal tumors through the use of a patient-specific 3-D-printed cutting guide: a feasibility study
Source: Pediatr Radiol. 2022 Aug 30;53(2):235–43. doi: 10.1007/s00247-022-05476-7 (PMC9892092; doi:10.1007/s00247-022-05476-7)
Supplement: Supplementary file 1 — Supplementary file1 (DOCX 652 kb) [file 247_2022_5476_MOESM1_ESM.docx]

**Online Supplementary Material 1** Patient-specific 3-D-printed cutting guides


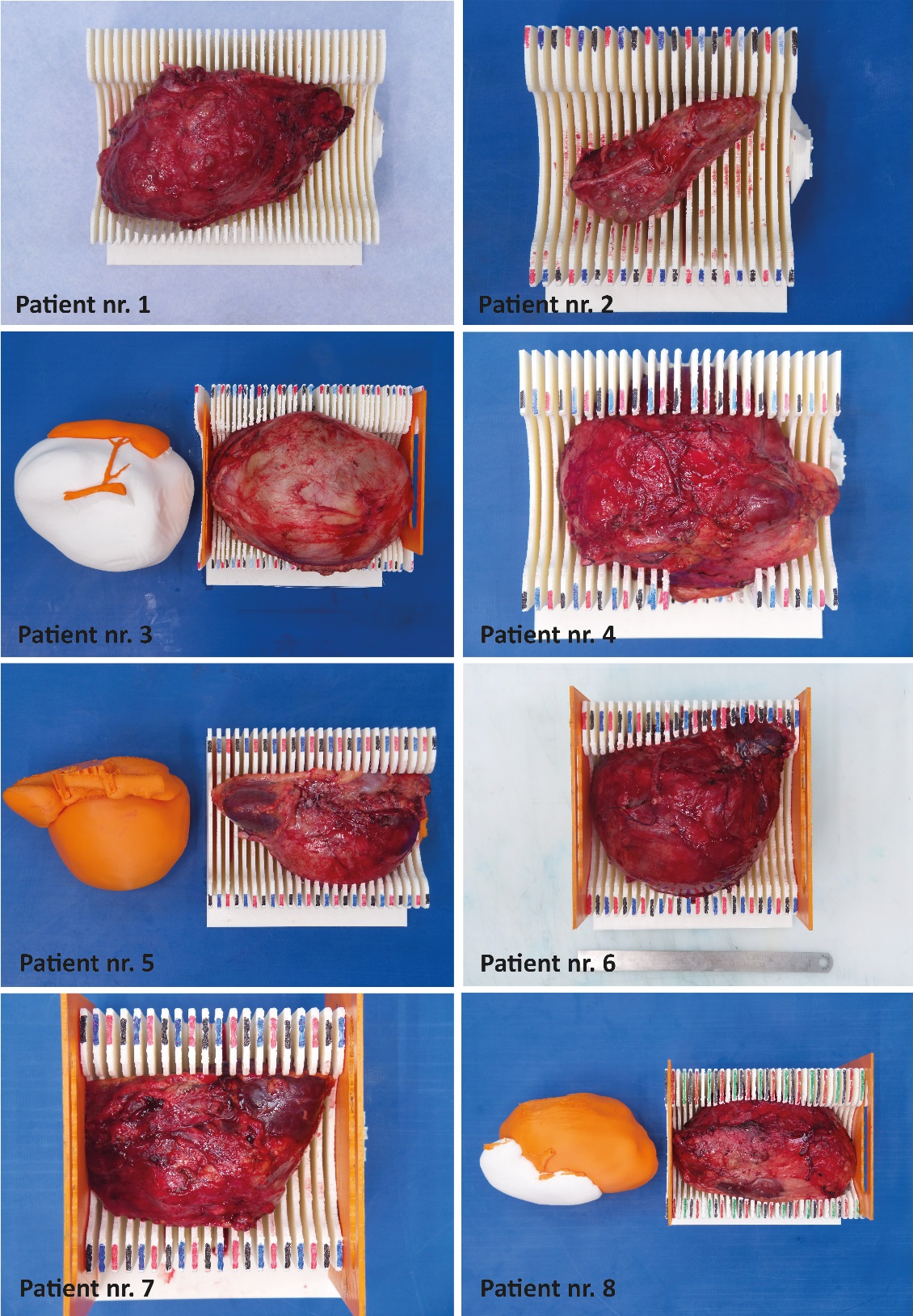


For each patient, a patient-specific 3-D-printed cutting guide was designed and printed based on his/her preoperative MRI scan. In some cases, there was enough time to also print the 3-D model of the kidney and tumor; however, this had no further consequences for the use of the cutting guide. The cutting guide barriers were alternately colored to support the pathologist in correctly positioning the knife. Moreover, the colors allowed for the repositioning of two movable support barriers to keep the specimen positioned during the slicing process.

*Nr* Number
