# Supplementary material for: Direct correlation of MRI with histopathology in pediatric renal tumors through the use of a patient-specific 3-D-printed cutting guide: a feasibility study
Source: Pediatr Radiol. 2022 Aug 30;53(2):235–43. doi: 10.1007/s00247-022-05476-7 (PMC9892092; doi:10.1007/s00247-022-05476-7)
Supplement: Supplementary file 2 — Supplementary file2 (DOCX 54.1 kb) [file 247_2022_5476_MOESM2_ESM.docx]

**Online Supplementary Material 2** Questionnaire feasibility study

**Questionnaire – Feasibility Study**

***Direct correlation of MRI with histopathology in pediatric renal tumors through the use of a patient-specific 3-D-printed cutting guide: a feasibility study***

Please answer this questionnaire during or as soon as possible after the use of the 3-D-printed patient-specific cutting guide for slicing the renal tumor. For each question, the responsible specialist is indicated. Surgeon Pathologist/Pathology-assistant PhD-student

**1. Information and characteristics**

**1.1 Study number**

**1.2 SIOP-number**

**1.3 Date of the surgery** (dd/mm/yy) //

**1.4 Weight of the specimen** *(gram)*

**1.4 Dimensions of the tumor** *(cm)* x x

**1.3 Questionnaire filled in by** Surgeon

Pathologist(-assistant)

Lead investigator

**2. Positioning of the kidney in the cutting guide and transport**

**2.1 On a scale from 1 to 5, the positioning of the kidney in the cutting guide in the OR was:**

| Very difficult | Difficult | Not difficult, not easy | Easy | Very easy |
| --- | --- | --- | --- | --- |
| 1 | 2 | 3 | 4 | 5 |

**2.2 What was the situation when arriving at the OR for the pick-up of the tumor?**

□ The kidney was already positioned in the cutting guide by the surgeon(s)

□ The kidney was positioned in the cutting guide after I arrived, together with/with the guidance of the surgeon(s)

□ I positioned the kidney in the cutting guide myself, without help/guidance of the surgeon(s)

Room for comment

**2.3 On a scale from 1 to 5, this approach was:**

| Totally not convenient | Not convenient | Not good, not bad | Convenient | Very convenient |
| --- | --- | --- | --- | --- |
| 1 | 2 | 3 | 4 | 5 |

**2.4 During my transfer with the kidney from the OR to the diagnostic laboratory, concerning the positioning of the kidney in the cutting guide, I:**

□ Have not experienced any problems

□ Have experienced minor problems

● Please specify:

□ Have experienced major problems

● Please specify:

**2.5 After taking the kidney out of the cutting guide to dye the specimen, I positioned the kidney in the cutting guide again with help of** *(you can choose multiple answers)*:

□ The picture taken of the kidney in the cutting guide.

□ My memory of the way the kidney was positioned in the cutting guide.

□ The fitting of the kidney in the cutting guide and the indications written on the cutting guide.

□ The illustration of the design of the cutting guide including the 3D-model of the kidne.y

□ Another pathologist and/or pathasser.

□ The appearance of the kidney on the MRI scan.

□ The PhD student(s).

□ A radiologist.

□ A surgeon.

Room for comment

**2.6 After taking the kidney out of the cutting guide to dye the specimen, I found, on a scale from 1 to 5, repositioning the tumor in the cutting guide (with, in case this was necessary, help (2.5)):**

| Very difficult | Difficult | Not difficult, not easy | Easy | Very easy |
| --- | --- | --- | --- | --- |
| 1 | 2 | 3 | 4 | 5 |

**3. Ease of use and feasibility of the cutting guide for the slicing of the kidney**

**3.1 On a scale from 1 to 5, the slicing of the kidney using the cutting guide was:**

| Very difficult | Difficult | Not difficult, not easy | Easy | Very easy |
| --- | --- | --- | --- | --- |
| 1 | 2 | 3 | 4 | 5 |

**3.2 During the slicing of the tumor, the following ‘flaws’ of the cutting guide made it less feasible** *(including, if applicable, the influence of the shape, consistency, composition, etc. of the tumor/specimen on the use of the cutting guide)***:**


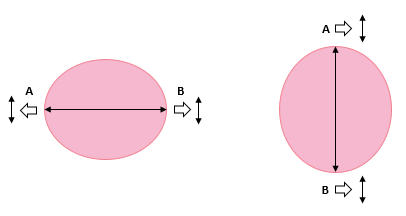
**4. Results of the use of the cutting guide**

**4.1 The thickness** *(in mm, no decimals)* **of each slice separately, measured on the opposite sites of the longest diameter** **is:**

| **Slice** | **Thickness** *(mm)* | | **Slice** | **Thickness** *(mm)* | | **Slice** | **Thickness** *(mm)* | |
| --- | --- | --- | --- | --- | --- | --- | --- | --- |
|  | A | B |  | A | B |  | A | B |
| **1** |  |  | **11** |  |  | **21** |  |  |
| **2** |  |  | **12** |  |  | **22** |  |  |
| **3** |  |  | **13** |  |  | **23** |  |  |
| **4** |  |  | **14** |  |  | **24** |  |  |
| **5** |  |  | **15** |  |  | **25** |  |  |
| **6** |  |  | **16** |  |  | **26** |  |  |
| **7** |  |  | **17** |  |  | **27** |  |  |
| **8** |  |  | **18** |  |  | **28** |  |  |
| **9** |  |  | **19** |  |  | **29** |  |  |
| **10** |  |  | **20** |  |  | **30** |  |  |

Room for comment

**4.2 The diameter** *(nr. slice, in cm, rounded down to one decimal)* **of 5 equally distributed slices to determine the feasibility of the slicing of the specimen in the cutting guide is:**

| Anterior – Posterior |  |
| --- | --- |
| Lateral – Medial |  |

| Anterior – Posterior |  |
| --- | --- |
| Lateral – Medial |  |

| Anterior – Posterior |  |
| --- | --- |
| Lateral – Medial |  |

| Anterior – Posterior |  |
| --- | --- |
| Lateral – Medial |  |

| Anterior – Posterior |  |
| --- | --- |
| Lateral – Medial |  |

**4.3 Were there slices that did not show the desired result due to a specific reason?**

□ No

□ Yes -> If yes, please indicate the number of the slice(s) and the specific reason that led to a discrepancy/unwanted result in this slice *(for example: ‘mistake while cutting’ or ‘displacement specimen in the cutting guide during process’)*

| **Slice number** | **Reason for discrepancy/undesired result** |
| --- | --- |
|  |  |
|  |  |
|  |  |
|  |  |
|  |  |
