# Supplementary material for: Direct correlation of MRI with histopathology in pediatric renal tumors through the use of a patient-specific 3-D-printed cutting guide: a feasibility study
Source: Pediatr Radiol. 2022 Aug 30;53(2):235–43. doi: 10.1007/s00247-022-05476-7 (PMC9892092; doi:10.1007/s00247-022-05476-7)
Supplement: Supplementary file 3 — Supplementary file3 (DOCX 15.3 kb) [file 247_2022_5476_MOESM3_ESM.docx]

**Online Supplementary Material 3** Details of the questionnaire-based qualitative analysis

| **Question** | **1** | **2** | **3** | **4** | **5** |
| --- | --- | --- | --- | --- | --- |
|  | **Very difficult** | **Difficult** | **Not difficult, not easy** | **Easy** | **Very easy** |
| Positioning of the specimen in the cutting guide | 0/8 | 0/8 | 1/8 | 2/8 | 5/8 |
| Repositioning of the specimen in the cutting guide after taking it out for standard clinical pathological workflow procedures | 0/8 | 0/8 | 0/8 | 4/8 | 4/8 |
| Slicing of the specimen in the cutting guide | 0/1 | 1/8 | 1/8 | 3/8 | 3/8 |
| **Question** | **1** | **2** | **3** | **4** | **5** |
|  | **Totally not convenient** | **Not convenient** | **Not good, not bad** | **Convenient** | **Very convenient** |
| Positioning of the specimen in the cutting guide by the lead investigator, guided by the surgeon | 0/8 | 0/8 | 0/8 | 3/8 | 5/8 |
