# Supplementary material for: Direct correlation of MRI with histopathology in pediatric renal tumors through the use of a patient-specific 3-D-printed cutting guide: a feasibility study
Source: Pediatr Radiol. 2022 Aug 30;53(2):235–43. doi: 10.1007/s00247-022-05476-7 (PMC9892092; doi:10.1007/s00247-022-05476-7)
Supplement: Supplementary file 4 — Supplementary file4 (DOCX 530 kb) [file 247_2022_5476_MOESM4_ESM.docx]

**Online Supplementary Material 4** Visualization of the line-width of 3-D printing material


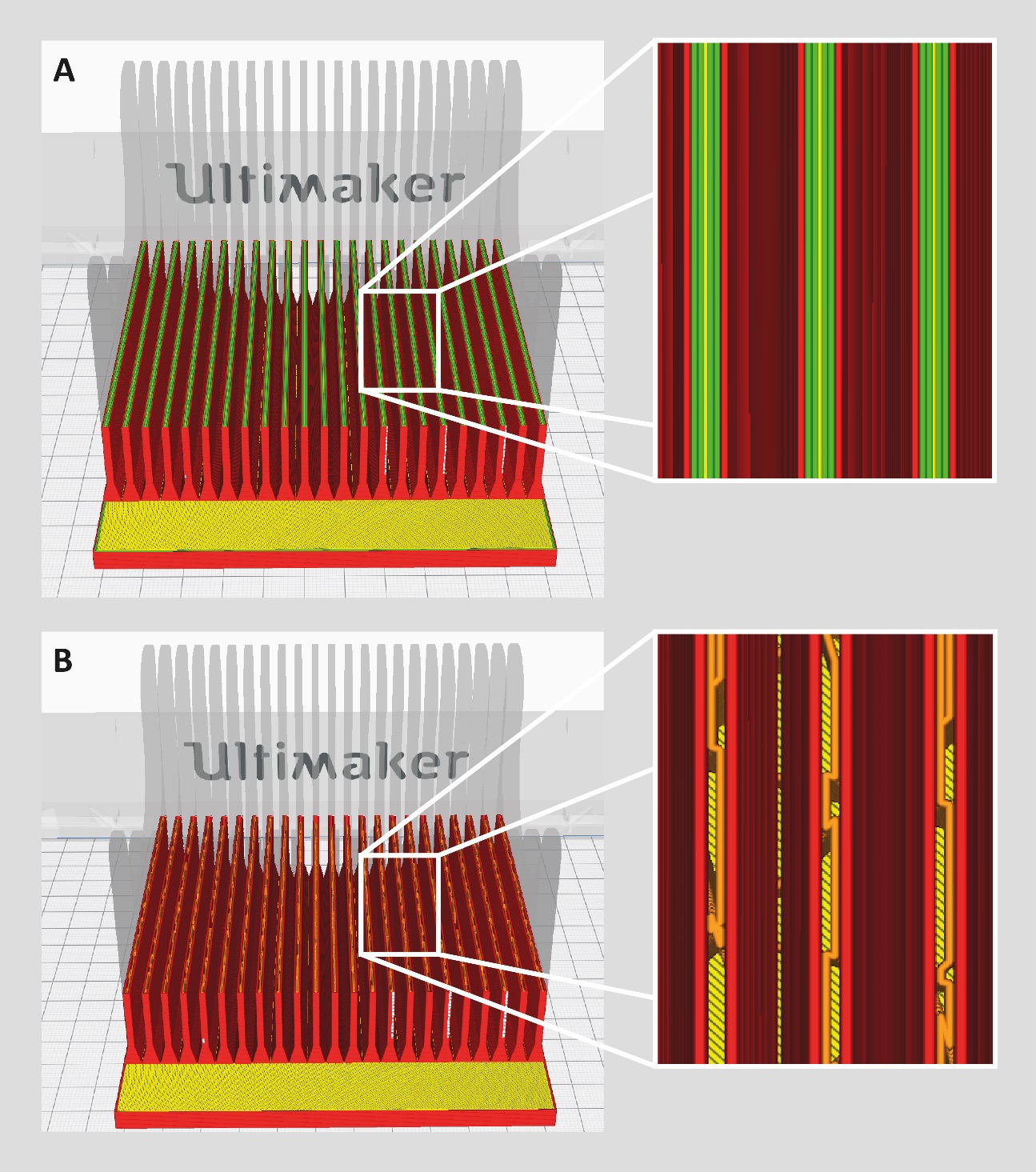


Visualization of the cutting guide with different 3-D printing settings (**a** and **b**), each line representing 3-D printing material, with a detailed visualization of three separate barriers. The outer wall of the model is shown in red, the inner wall in green, the infill in orange and a top layer in yellow.

**a** The cutting guide barriers with regular settings: a line-width of 0.2 mm and a wall-line count of 3. The barriers are completely filled with 3-D printing material resulting in long printing times.

b The cutting guide with an increased line width of 0.6 mm and a reduced wall-line count of 1. This requires less material and decreased printing time; the triangular structured infill material (orange) ensures stability of the barrier. This enabled a higher printing quality without increasing the printing time
